# Hypofractionated radiation therapy and wound healing after massive sarcoma resection: Case report and review of the literature

**DOI:** 10.1016/j.ijscr.2021.106005

**Published:** 2021-05-21

**Authors:** Michael Allen, Daniella Silvino, Mitchell Kamrava, Wonwoo Shon, Earl Brien

**Affiliations:** a Department of Orthopaedic Surgery, Community Memorial Health System, 147 N Brent St. Ventura, CA 93003, United States of America; b NYIT College of Osteopathic Medicine, 101 Northern Blvd, Glen Head, NY 1145, United States of America; c Department of Radiation Oncology, Cedars Sinai Medical Center, 8700 Beverly Blvd, Los Angeles, CA 90048, United States of America; d Department of Pathology and Laboratory Medicine, Cedars Sinai Medical Center, 8700 Beverly Blvd, Los Angeles, CA 90048, United States of America; e Department of Orthopaedic Surgery, Cedars Sinai Medical Center, 8700 Beverly Blvd, Los Angeles, CA 90048, United States of America

**Keywords:** Case report, Sarcoma, Hypofractionated radiation, Liposarcoma, Myxofibrosarcoma

## Abstract

**Introduction:**

Large high-grade sarcomas are commonly managed with five weeks of pre-operative radiation with chemotherapy followed by surgical resection. Wound complications occur in about one out of three patients with this regimen. Hypofractionated radiation therapy (HFRT) is a developing pre-operative approach that delivers radiation over a shorter duration of 5–10 treatments.

**Presentation of case:**

Two patients underwent HFRT with neoadjuvant chemotherapy followed by tumor resection. The first patient had high-grade de-differentiated liposarcoma, and the second patient a high-grade myxofibrosarcoma. Neither patient developed post-operative wound complications despite the massive tumor size.

**Discussion:**

Less is understood regarding rates and risk factors associated with wound complications using this shortened radiation approach. With attention to surgical detail, and advancing radiation delivery technologies, rates of complications can be minimized.

**Conclusion:**

We discuss our experience with a neoadjuvant hypofractionated chemoradiation protocol in two patients with large volume sarcomas resected from the chest wall and the thigh who did not develop acute wound complications. Further evaluation of this shortened regimen is warranted.

## Highlights

• Wound complications are common after sarcoma resection with preoperative radiation.
• Hypofractionated Radiation Therapy (HFRT) may be a safe alternative to conventional dose radiation.
• HFRT can be utilized with massive volume sarcomas safely.

## 1 Introduction

Sarcomas are uncommon malignant tumors of mesenchymal origin, 80% of which originate from soft tissues [1]. The standard therapy involves surgical resection and radiation therapy (RT) to minimize local recurrence risk with or without chemotherapy. RT can be delivered pre- or post-operatively with equal oncologic efficacy but varying toxicities [2]. One of the most concerning side effects of preoperative RT is wound healing complications. Efforts to understand factors associated with higher rates of wound complications following preoperative RT are ongoing but include factors like tumor size, tumor location, tumor proximity to the skin, and medical co-morbidities [[2], [3], [4], [5], [6], [7]]. There is growing interest in reducing the time it takes to deliver preoperative RT using more HFRT regimens (8 or 5 treatments) [8,9]. Whether these shorter regimens have similar wound complication rates as standard RT is not well known, especially for larger tumor volumes. We report the cases of two patients who successfully received neoadjuvant HFRT to treat massive soft tissue sarcomas without wound healing complications.

## 2 Case report

This work is reported in line with the SCARE criteria [10].

**Fig. 1:**
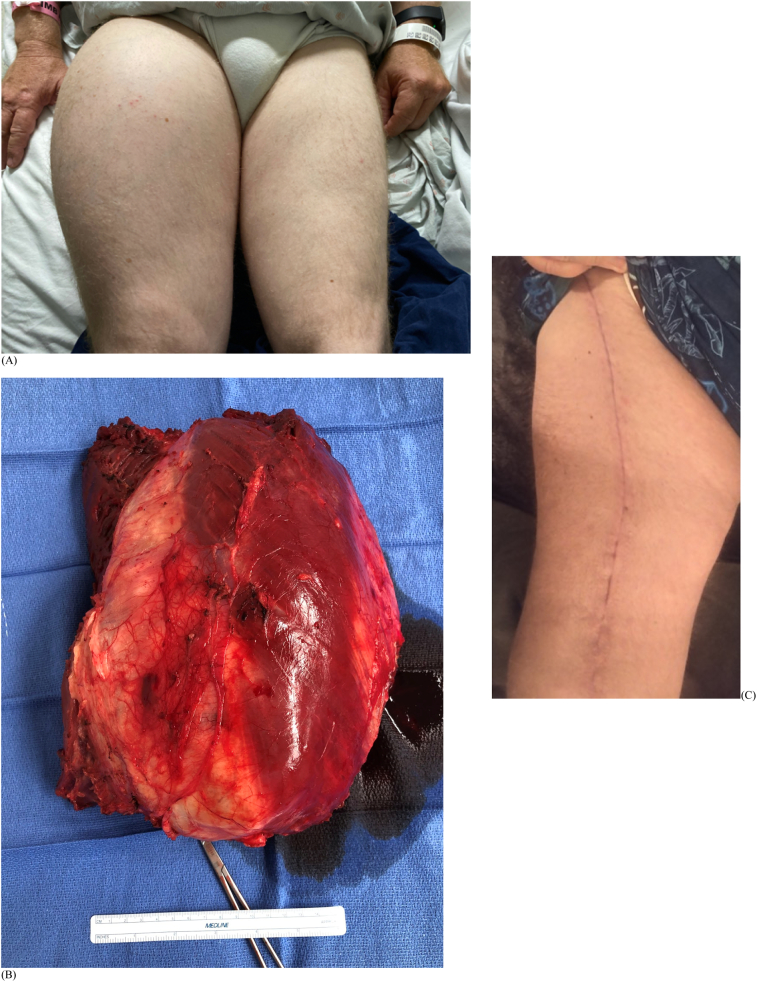
Patient 1 (A) initial presentation with right thigh mass; (B) tumor resection specimen; (C) surgical scar at 10 weeks.

**Fig. 2:**
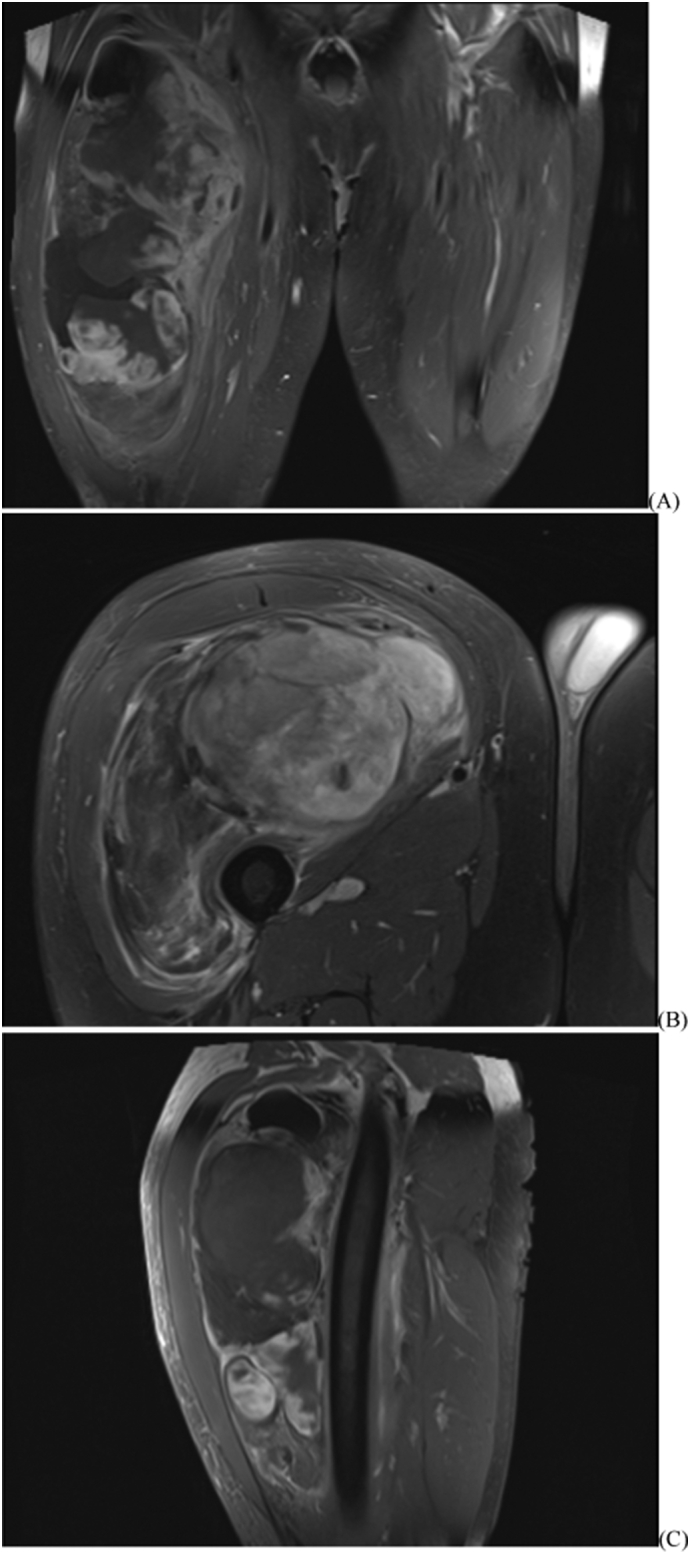
Patient 1 post-contrast MRI of right thigh tumor. (A) T1 fat-saturated coronal; (B) proton density fat-saturated axial; (C) T1 fat-saturated sagittal.

**Fig. 3:**
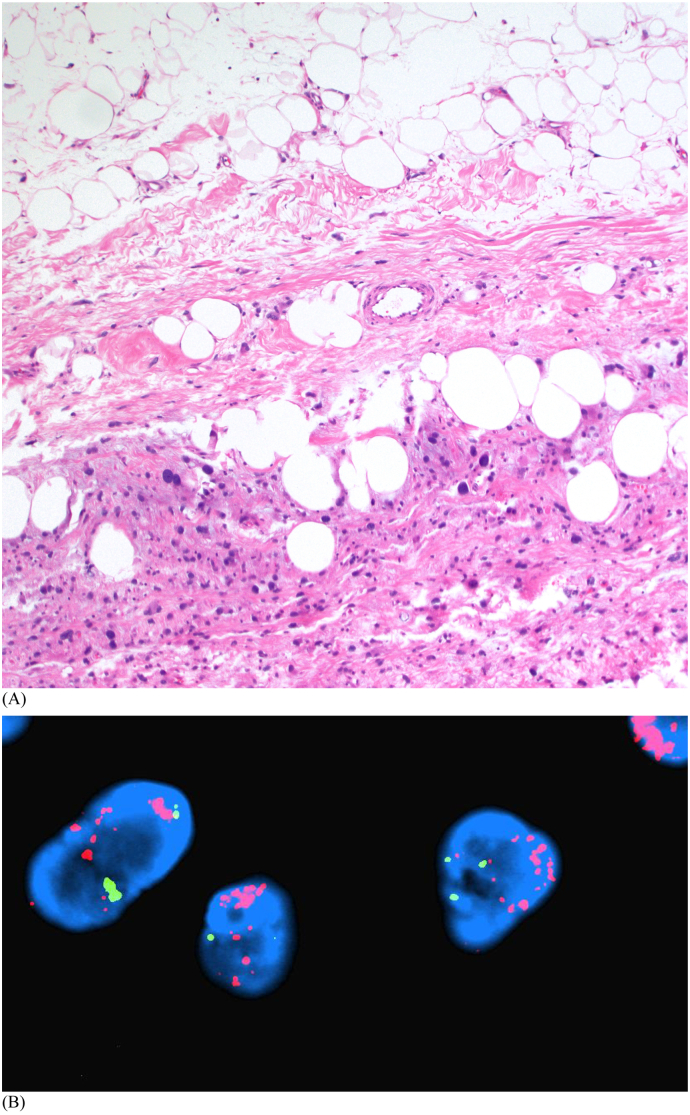
Abrupt transition from well-differentiated liposarcoma to high-grade dedifferentiated liposarcoma (A; H&E ×100). By Fluorescence in situ hybridization, the tumor cells showed high-level MDM2 gene amplification (B); MDM2 red and CEP12 green. (For interpretation of the references to colour in this figure legend, the reader is referred to the web version of this article.)

Patient 1 is a 62-year-old male who noticed a right thigh mass after difficulty putting on a pair of trousers (Fig. 1). He had no pain or neurological symptoms, but noted continued enlargement. The patient had no pertinent medical history. A deep, firm and immobile mass in the anterior thigh measured roughly 25 cm by 15 cm on physical exam. There was no skin compromise, palpable inguinal lymphadenopathy, or neurovascular deficits. MRI showed a complex mass deep to the vastus and adductor musculature, measured 26 cm in length, 17 cm in width, and 10 cm in depth. The tumor encircled the anterior femur without underlying bony signal changes. A relatively low signal mass was seen on T1 weighted images and a heterogeneous bright signal on T2 weighted images (Fig. 2). A CT-guided biopsy of the right thigh demonstrated a high-grade dedifferentiated liposarcoma with MDM2 gene amplification (Fig. 3).

After multi-disciplinary discussion, the patient began neoadjuvant Adriamycin and ifosfamide for 2 cycles, followed by HFRT with concurrent ifosfamide infusion as per Pennington et al. [9]. Target delineation was based on the recommendations from RTOG 0630 [7]. He received 28 Gy over eight fractions using intensity-modulated radiation therapy (IMRT) and daily image-guided radiation therapy delivery. Early radiation side effects were limited to Grade 1 skin changes. One month after completing therapy, he underwent radical resection of the tumor by the senior author. Surgical technique involved raising fasciocutaneous flaps followed by circumferential elevation of the anterior musculature and periosteum, sparing the rectus femoris. The femoral artery, vein, and saphenous nerve were skeletonized and protected. The tumor was removed en bloc and measured 28.1 × 19.5 × 11.2 cm (Fig. 1). Intraoperative margins were negative for tumor. Primary layered closure followed, over a deep drain removed at the 2-week follow-up. Histologically the tumor was Grade 3 with 30% necrosis, one mitosis per 10 high-powered fields. The closest margin was 1 mm. Postoperative recovery was uneventful. At 90 days, the surgical wound healed without wound complications or early toxicity (Fig. 1). Postoperative radiation was not performed at the request of the patient after discussion with the radiation oncologist. The patient also elected not to proceed with adjuvant chemotherapy and will be monitored with repeat surveillance imaging.

**Fig. 4:**
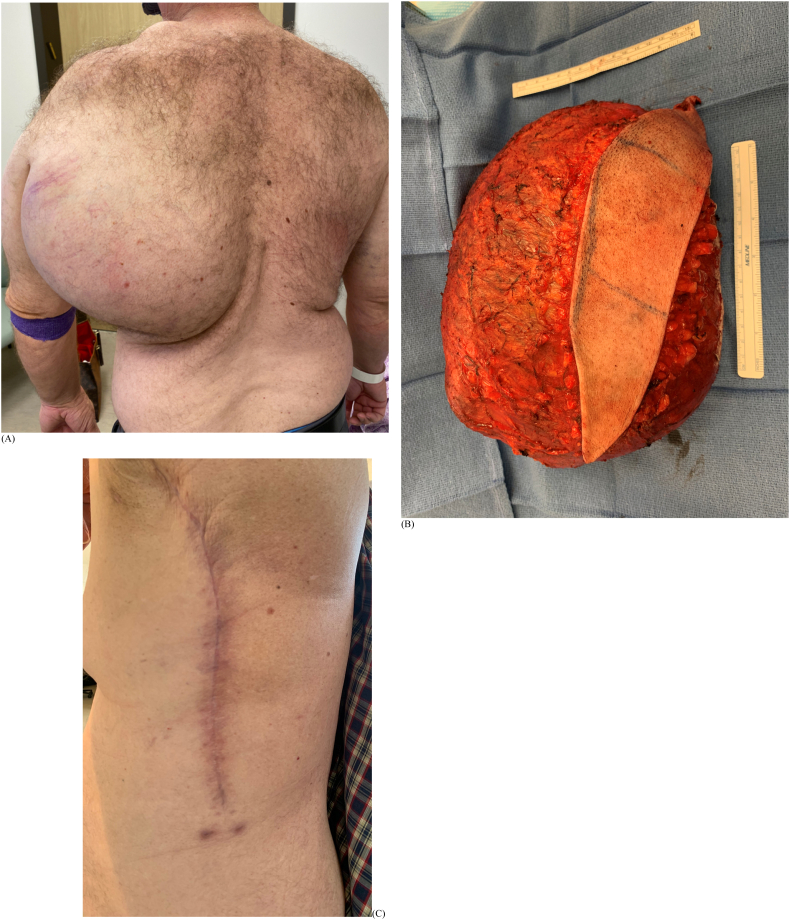
Patient 2 (A) initial presentation with right axilla/chest wall mass; (B) tumor resection specimen; (C) surgical scar at 10 weeks.

**Fig. 5:**
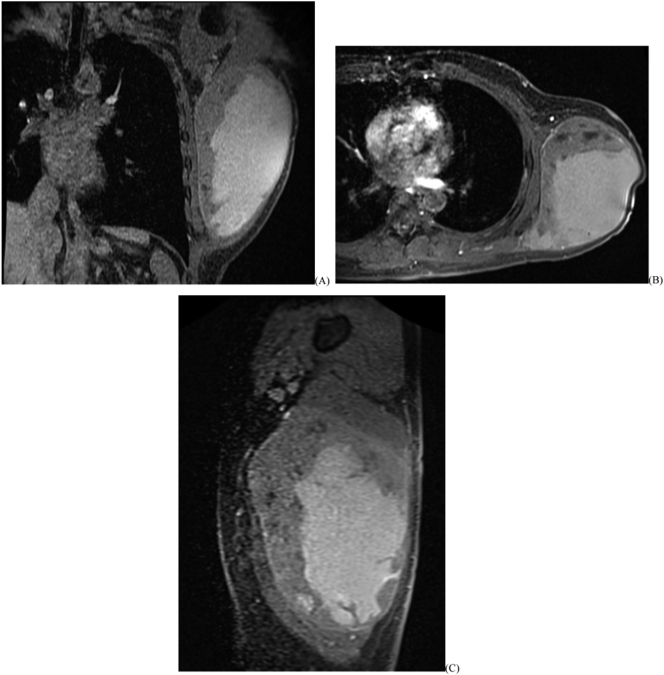
Patient 2 T1 fat-saturated MRI; (A) coronal; (B) axial; (C) sagittal.

**Fig. 6:**
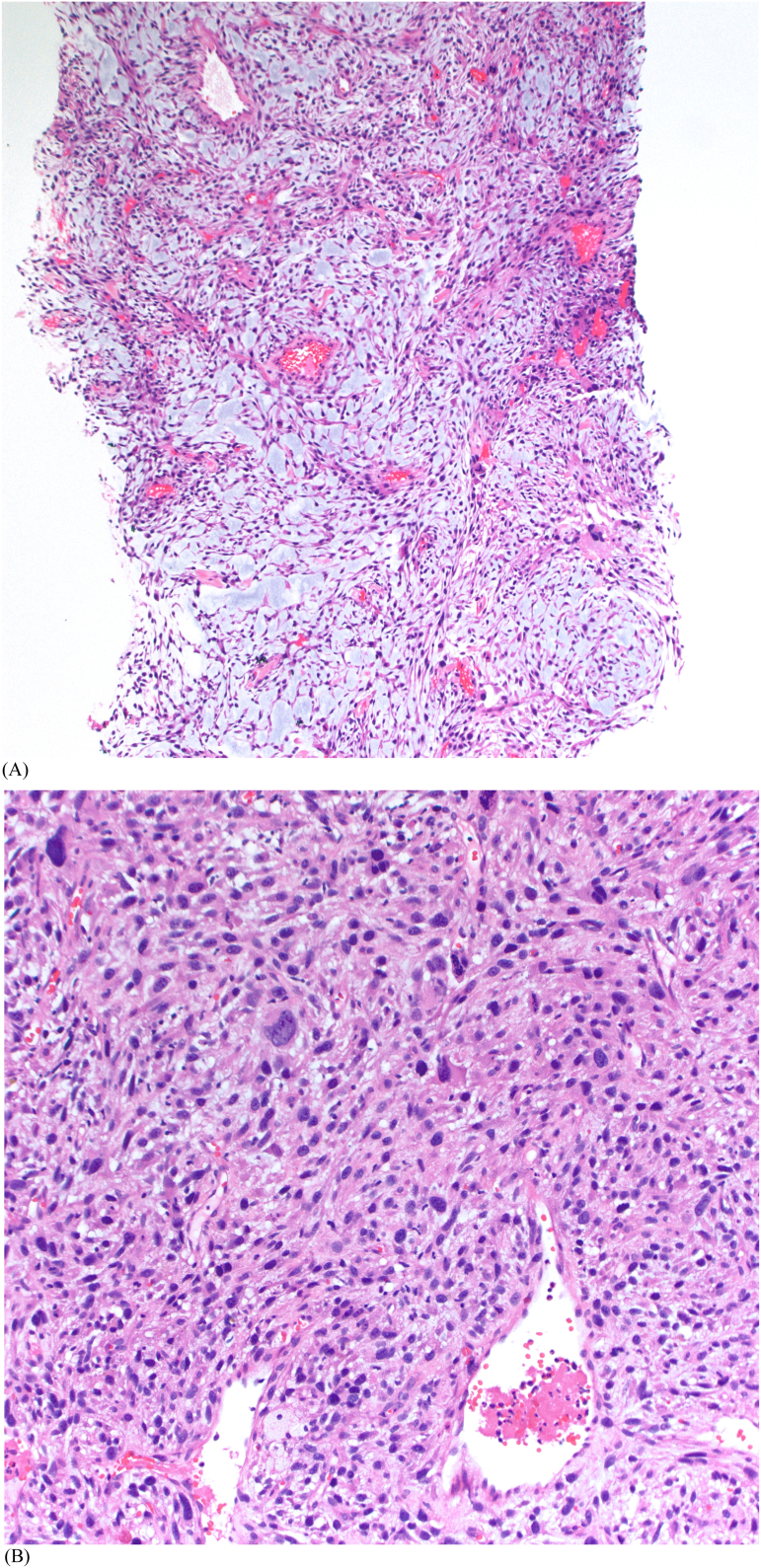
Myxofibrosaracoma with prominent myxoid stroma and characteristic curvilinear vessels (A, H&E ×100). At high magnification, the tumor cells show significant nuclear pleomorphisms and mitotic activity (B, H&E ×200).

Patient 2 is a 58-year-old male who presented six months after noticing an enlarging mass in the left axilla (Fig. 4). The mass became progressively more painful with increasing pressure but no neurological symptoms in the arm. An outside provider aspirated over 500 cc brown fluid from the mass before the initial Orthopaedic examination. The patient had no pertinent medical history. Exam demonstrated a 22 cm × 20 cm mass, tender to palpation, deep to the latissimus dorsi extending into the axilla. There were no overlying skin changes, and he had full function of the arm without deficits. MRI demonstrated a mass 25.5 cm in length, 11 cm wide, and 11 cm deep, located on the left chest wall deep to the latissimus dorsi (Fig. 5). The proximal apex of the tumor was adjacent to the axillary vasculature and brachial plexus. On T2 sequences, the mass showed heterogeneous signal, septations, and central fluid collection. CT-guided biopsy demonstrated high-grade myxofibrosarcoma (Fig. 6).

We pursued the same regimen as Patient 1, with two rounds of neoadjuvant Adriamycin with ifosfamide followed by 28 Gy given in eight fractions with IMRT with concurrent ifosfamide therapy. He tolerated chemoradiation with only Grade 1 acute skin changes. One month after completing therapy, radical resection of the mass by the senior author followed. The mass was removed en-bloc with skin ellipse after long thoracic nerve neurolysis, and axillary dissection. The specimen measured 26 × 19.5 × 13 cm (Fig. 4). Drain was removed at the 2-week follow-up. Histology revealed grade 3 myxofibrosarcoma with 85% necrosis, 0 mitoses per 10 high-power fields, and the closest margin was 1.5 mm. Intraoperative margins were free of malignancy. Postoperative recovery was uneventful without wound complications or early toxicity (Fig. 4). The patient elected to continue with adjuvant Adriamycin and ifosfamide therapy but no additional radiation.

## 3 Discussion

We treated two patients with substantial volume high-grade sarcomas with HFRT and concurrent chemotherapy without acute wound complications. We believe attentive surgical technique including the development of local flaps, meticulous closure, and IMRT with image-guided radiation therapy, may have helped minimize wound complications even when treating larger tumors with a shorter course of preoperative RT.

The use of radiation or chemoradiation followed by surgical resection is the standard of care for large high-grade soft tissue sarcomas [11]. Preoperative RT advantages include using a lower dose, a smaller treatment field, and easier target delineation, though with increased risk of acute wound complications. The landmark National Cancer Institute of Canada SR2 randomized trial by O'Sullivan et al., which delivered a dose of 50 Gy in 2 Gy fractions, reported a wound complication rate of 35%. Improvements in radiation delivery techniques with IMRT, image-guided radiation therapy, and smaller margins demonstrate significant reductions in late complications at two years but similar rates of acute wound complications with the SR2 trial [5,6]. Attempts to improve acute wound complication rates by reducing the radiation dose to uninvolved tissues show promise but without significant differences compared with the SR2 trial [12].

A Phase 2 study of a five-treatment preoperative RT regimen showed similar wound complications compared with the SR2 study and similar local control rates [8]. Complications were not associated with tumor size, depth, or dose at the skin. In another five-treatment preoperative RT study, Kosela et al. prospectively reported 272 patients who underwent surgical resection within seven days of completing HFRT [13]. This series had an 11.8% rate of wound dehiscence and a 16.5% rate of prolonged healing. Notably, in this series, only 7% of patients required reoperation for wound complication compared to 16% in the SR2 trial [2,13]. A similar protocol by Parsai et al. delivered around 30 Gy over five fractions (27.5–40 Gy) followed by resection within seven days [14]. In this series of 16 patients, the wound complication rate was 31% overall, with 19% requiring reoperation. Further understanding of whether this regimen can be used on all preoperative patients remains to be determined, or whether tumor size/location/use of chemotherapy should factor into an ideal candidate for five versus 25 treatment regimens.

There is limited data on whether similar risk factors are associated with wound complications in HFRT compared with the more standard 25 treatment regimen. In the most extensive report of the 8-treatment regimen, acute wound complications occurred in 11% of patients [9]. While about 50% of the patients on this study had tumors >10 cm, the mean tumor size was not reported, so it is unclear if this regimen has similar or different toxicity in massive tumors over 20 cm. With a ten treatment preoperative regimen with concurrent chemotherapy, a wound complication rate of 20% was reported [15]. This demonstrates a variation in wound complication data from various series [11,12]. Studies suggest tumor size >10 cm and proximity <3 mm from skin are significant risk factors, along with diabetes, associated with increased risks of acute wound complications [4]. Several studies have shown a higher association of wound complications with tumors located in the lower extremity, specifically in the thigh [[2], [3], [4], [5], [6], [7]].

As interest and data increase for using shorter preoperative RT regimens, an improved understanding of ideal candidates and predictors of wound complications is very much needed. Randomized prospective studies are needed to compare the short-term effectiveness and wound complication rates and the long-term toxicities and survival rates between standard and HFRT.

## 4 Conclusion

In conclusion, HFRT is becoming more common in treating sarcomas and has the potential advantages of increased accessibility and lower social demands. Our two patients had large tumors, measured by pathology to be around 6137 mL and 6591 mL at the time of resection, and underwent an 8-treatment preoperative RT regimen with concurrent chemotherapy. Despite the size and shortened preoperative RT regimen, neither developed acute wound complications. Our results are encouraging and suggest further evaluation of this shorter preoperative regimen.

## Provenance and peer review

Not commissioned, externally peer-reviewed.

## Sources of funding

None.

## Ethical approval

IRB approval was waived for this case report per the institutional IRB protocols.

## Consent

Written informed consent was obtained from the patient for publication of this case report and accompanying images. A copy of the written consent is available for review by the Editor-in-Chief of this journal on request.

## Author contribution

Michael Allen: Writing original draft, visualization, project administration;

Daniella Silvino: Writing original draft

Mitchell Kamrava: Writing review & editing, conceptualization, validation, investigation;

Wonwoo Shon: visualization, resources

Earl Brien: Conceptualization, methodology, supervision, investigation.

## Research registration

N/A.

## Guarantor

Michael Allen DO.

## Declaration of competing interest

None declared.
